# Gut bacterial metabolites modulate endoplasmic reticulum stress

**DOI:** 10.1186/s13059-021-02496-8

**Published:** 2021-10-15

**Authors:** Xiaobo Ke, Kwontae You, Matthieu Pichaud, Henry J. Haiser, Daniel B. Graham, Hera Vlamakis, Jeffrey A. Porter, Ramnik J. Xavier

**Affiliations:** 1 Broad Institute of MIT and Harvard, Cambridge, MA 02142 USA; 2 Novartis Institute for Biomedical Research Inc., Cambridge, MA 02139 USA; 3 Center for Computational and Integrative Biology and Department of Molecular Biology, Massachusetts General Hospital, Harvard School of Medicine, Boston, Massachusetts 02114 USA; 4 Center for Microbiome Informatics and Therapeutics, Massachusetts Institute of Technology, Cambridge, MA 02139 USA

**Keywords:** ER stress, Unfolded protein response, Microbial metabolites, Intestinal epithelial homeostasis

## Abstract

**Background:**

The endoplasmic reticulum (ER) is a membranous organelle that maintains proteostasis and cellular homeostasis, controlling the fine balance between health and disease. Dysregulation of the ER stress response has been implicated in intestinal inflammation associated with inflammatory bowel disease (IBD), a chronic condition characterized by changes to the mucosa and alteration of the gut microbiota. While the microbiota and microbially derived metabolites have also been implicated in ER stress, examples of this connection remain limited to a few observations from pathogenic bacteria. Furthermore, the mechanisms underlying the effects of bacterial metabolites on ER stress signaling have not been well established.

**Results:**

Utilizing an XBP1s-GFP knock-in reporter colorectal epithelial cell line, we screened 399 microbiome-related metabolites for ER stress pathway modulation. We find both ER stress response inducers (acylated dipeptide aldehydes and bisindole methane derivatives) and suppressors (soraphen A) and characterize their activities on ER stress gene transcription and translation. We further demonstrate that these molecules modulate the ER stress pathway through protease inhibition or lipid metabolism interference.

**Conclusions:**

Our study identified novel links between classes of gut microbe-derived metabolites and the ER stress response, suggesting the potential for these metabolites to contribute to gut ER homeostasis and providing insight into the molecular mechanisms by which gut microbes impact intestinal epithelial cell homeostasis.

**Supplementary Information:**

The online version contains supplementary material available at 10.1186/s13059-021-02496-8.

## Background

Endoplasmic reticulum (ER) stress contributes to various human diseases including inflammatory conditions, cancer, neurodegeneration, fibrosis, diabetes, and metabolic disorders [1–3]. Preclinical models indicate that manipulation of specific ER stress mediators may have beneficial or detrimental effects on the severity of various diseases depending on the specific disease context [4–9]. As a fundamental component of the secretory pathway, the ER plays an essential role in protein folding and maturation (including *N*-glycosylation, and intra- and intermolecular disulfide bond formation), in addition to other metabolic processes including lipid biosynthesis, gluconeogenesis, and mitochondrial bioenergetics. Accumulation of misfolded proteins and ER stress can derive from excessive secretory demands, loss of calcium balance, lipid toxicity, and expression of disease-related mutant proteins. To counteract ER stress, cells engage in the unfolded-protein response (UPR). The UPR consists of three parallel signal-transduction cascades that reprogram the cell to augment ER chaperone expression, facilitate misfolded protein elimination, and halt general protein translation.

The UPR incorporates information about intensity and duration of the stress stimuli to restore proteostasis or trigger apoptosis of the irreversibly damaged cells. This is initiated by three transmembrane stress sensors: inositol-requiring enzyme 1 (IRE1α and IRE1β), protein kinase RNA-like ER kinase (PERK) and activating transcription factor 6 (ATF6). IRE1α contains a serine/threonine kinase and endoRNase (RNase) domain on its cytosolic region, which is sequestered by the ER-localized chaperone BiP (binding immunoglobulin protein) under physiological conditions [10]. In response to ER stress, binding of unfolded proteins to BiP releases IRE1α to self-dimerize and autophosphorylate, causing a conformational change that activates the RNase domain. This RNase catalyzes removal of a 26-nucleotide intron within the nascently transcribed *XBP1* mRNA (*XBP1u*). The altered reading frame in the spliced mRNA (*XBP1s*) allows expression of the functional XBP1 variant (XBP1s), a potent transcription factor that regulates genes important in protein folding quality control and lipid synthesis [11, 12]. Similar to IRE1, PERK is also activated by autophosphorylation following disengagement from BiP. PERK phosphorylates the eukaryotic initiation factor-2 alpha (eIF2α), resulting in arrested protein translation and reduced protein-folding load in the ER [13, 14]. Phosphorylation of eIF2α also influences transcription of a subset of genes via ATF4, promoting the production of specific ER-resident proteins important for restoration of ER homeostasis. The third UPR arm involves ER stress-induced cleavage of membrane-bound ATF6 [15], releasing its N-terminal cytoplasmic domain for translocation into the nucleus, activating target genes such as *HSPA5* (which encodes BiP). ATF6 pathway activation also increases *XBP1* transcription [16], making XBP1s a critical node in the UPR response. While the UPR engages in restoration of ER homeostasis, paradoxically, its prolonged activation can result in apoptosis. One of the key proapoptotic responses involves PERK-eIF2α-ATF4 mediated production of CHOP (CCAAT enhancer binding protein homologous protein), a transcription factor encoded by *DDIT3* [17].

The secretory capacity of the gut epithelium, especially mucin and antimicrobial protein production, likely demands maintenance of ER proteostasis. Hypomorphic mutations in UPR genes have been genetically associated with secretory epithelium and specialized functions in inflammatory bowel diseases (IBD) [18–20]. *Xbp1* deletion in mouse intestinal epithelial cells resulted in Paneth and goblet cell apoptosis, spontaneous ileal inflammation, and increased sensitivity to dextran sodium sulfate (DSS)-induced colitis [18]. In addition, genetic knockout of *Ire1-β*, the isoform of Ire1 expressed in colonic and gastric epithelial cells, increased sensitivity to DSS-induced colitis [21]. By contrast, deletion of *Ddit3* in mice leads to decreased susceptibility to DSS-induced colitis, partially by restricting the amount of ER stress-induced apoptosis in intestinal epithelial cells [22]. The emerging evidence points toward the important contributions of ER stress to intestinal inflammation, but the mechanisms underlying ER stress in IBD patients are poorly understood.

Both the gut microbiota and metabolome are altered in IBD patients [23–25]. Microbes, microbial products, and their interactions likely influence ER stress in the gut. Gut microbiota interact with eukaryotic host cells through metabolites generated by a combination of de novo synthesis and secondary transformation of dietary and non-dietary precursors [26, 27]. Some microbial products are also known to modulate ER stress. For example, bacterial toxins (such as the Shiga toxins, VacA, and Listeriolysin O) induce ER stress and UPR signaling [28–31]. In addition, microbial-derived tunicamycin (Tm) is the most commonly deployed experimental inducer of ER stress. Tm blocks N-glycosylation and causes misfolding of many proteins in the ER. By contrast, Streptomyces polyketides (trierixin, mycotrienin II, and trienomyxin A) and Actinomycete metabolite (toyocamycin) are potent inhibitors of ER stress-induced XBP1 activation [32, 33]. Still, the full breadth of microbial metabolites that interact with ER stress signaling, as well as mechanisms by which these molecules impact this pathway, remains undefined. In this study, we aimed to uncover gut microbiome-related metabolites that could influence ER stress or the UPR. We found both inducers and suppressors of this response and characterized their activities on UPR gene transcription and translation.

## Results

### An XBP1s-GFP screen to identify microbiome molecules with UPR-modulating activity

Gut metabolites are jointly derived from diet, modified human metabolites and microbially derived compounds that act as key mediators between the gut microbiome and host biology. For example, short chain fatty acids (SCFAs) such as butyrate, acetate, and propionate are produced by gut bacteria and modulate host cell functions such as histone deacetylase (HDAC) activity, gene expression, cell proliferation, and immune response. Commensal microbes can also alter pools of available host-generated metabolites such as tryptophan derivatives. Previous work from our group has identified differences in fecal and serum metabolites in the context of IBD [25]. To investigate how these metabolites impact mucosal homeostasis and inflammation, we selected a library of 399 molecules (Additional file 1, Table S1). The library includes 37 amino acid derivatives and 32 short chain fatty acid derivatives predicted from metagenomes/KEGG mapping, 93 bile acids with structures similar to cholic acid, 118 metabolites that were differently abundant between the stool samples of IBD patients and healthy individuals, as well as 119 bacterial derived metabolites. We henceforth refer to these molecules as microbiome box (MBB) compounds.

**Fig. 1:**
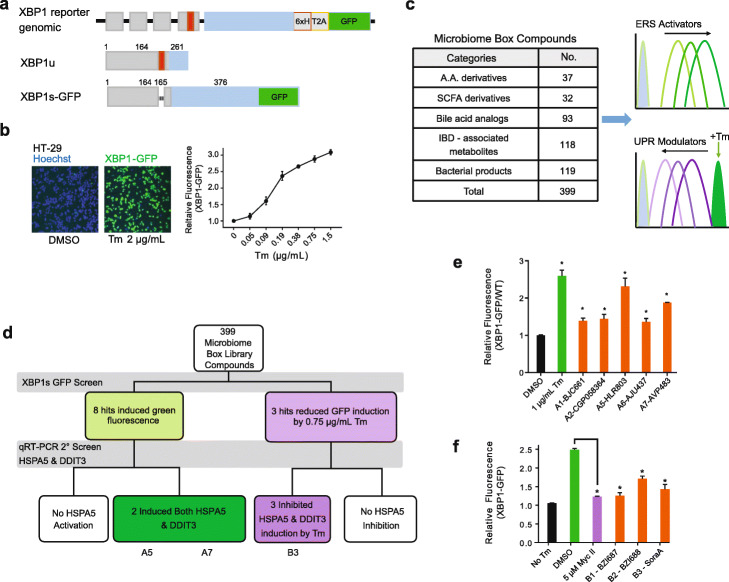
XBP1s-GFP screen identifies microbiome molecules with ER stress-modulating activities. **a** The XBP1 knock-in reporter construct in HT-29 cells encodes the 261 amino-acid (a.a.) XBP1u protein in homeostatic conditions. ER stress induces splicing of a 26-nucleotide fragment (red region) within XBP1 mRNA and leads to the production of 376 a.a. XBP1s protein and self-cleavable GFP. **b** Green fluorescence in HT-29 XBP1s-GFP reporter cells treated with DMSO or tunicamycin (Tm) was visualized by high content imaging. Relative fluorescence of XBP1s-GFP reporter cells in the presence of the specified Tm concentrations were normalized to DMSO-treated cells (0.5% v/v). **c** A library of chemical compounds (the “microbiome box”) relevant to microbiome and IBD were curated and screened for ER stress activation or dampened UPR response in the presence of Tm. **d** The screen identified eight compounds that induce fluorescence and three others that inhibit XBP1s-GFP. Validation by *HSPA5* and *DDIT3* mRNA expression confirmed activity of three molecules (A5, A7, and B3). **e** Five molecules specifically increased fluorescence in XBP1s-GFP reporter cells when supplemented at 50 μM, indicated by ratio of fluorescence in the reporter lines to parental HT-29 (XBP1s-GFP/WT). **f** Fluorescence induction by 0.75 μg/mL Tm (relative to no Tm treatment) in XBP1s-GFP reporter cells was measured when supplemented by DMSO, Myc II (positive control) or 50 μM of UPR inhibitors. In all bar graphs, error bars represent standard errors of the mean from three experimental replicates, and one-way ANOVA was used for statistical analyses (asterisks represent *P* < 0.05)

To monitor ER stress responses, we measured XBP1s since all three UPR signaling pathways upregulate XBP1s expression upon protein-folding stress. We generated an XBP1s-GFP knock-in reporter in a human colorectal adenocarcinoma cell line (HT-29) by CRISPR gene editing (Fig.1a). HT-29 in vitro culture is commonly used to model absorption, transport, and secretion by intestinal cells. As expected, addition of 2 μg/mL Tm (~ 2 μM) induced fluorescence by 3-fold in comparison to DMSO control after 12 h of incubation (Fig. 1b). The GFP induction by Tm is dose dependent with an EC_50_ of 0.2 μg/mL (Fig. 1b). This knock-in XBP1s-GFP reporter enables screening for molecules that modulate the ER stress pathway, as single-cell fluorescence can be measured in 384-well titer plates by high-content imaging and CellProfiler image analysis. We conducted two screens to identify ER stress induction activities, or ability to dampen UPR response induced by Tm treatment, using the MBB compounds (Fig. 1c).

We investigated the ability of MBB compounds to activate ER stress at four concentrations (50 μM, 15 μM, 5 μM to 1.5 μM), as indicated by an increase in green fluorescence of the XBP1s-GFP cells 12 h after compound treatment. Eight compounds were identified to induce fluorescence (Fig. 1d). For signal quantification, fluorescence was measured in the XBP1s-GFP cell line as well as the parental HT-29 cell line, which represents a baseline control. The ratio of fluorescence in the two cell lines in response to compound treatment (XBP1s-GFP/WT) therefore indicates the increase in fluorescence specifically due to XBP1s expression, correcting for chemical autofluorescence. As expected, the addition of 0.5% v/v DMSO does not induce relative fluorescence (Fig. 1e), whereas the addition of 1 μg/mL Tm activated relative fluorescence by 2.5-fold. Five of the 8 hit compounds (A1, A2, A5, A6, A7) were considered active (Fig. 1e, Additional file 2, Fig. S1a). Intriguingly, A1 (7-dehydrocholesterol), A2 (carnosol), and A6 (epoxysqualene) were found in our previous studies to be decreased in the stool of IBD patients compared to that from healthy individuals [25]. A5 and A7, two bacterial metabolites that induced the strongest response in the XBP1s-GFP cells after 12 h also resulted in a similar response after 36 hrs incubation (Additional file 2, Fig. S1b). None of the molecules affected HT-29 cell viability as measured by CellTiter-Glo assay, except for A7 treatment at 50 μM, which reduced viability by 50%.

Since activation of terminal UPR by ER stressors could lead to apoptosis and inflammation, we rationalized that dampening down the UPR response using bacterial-derived molecules could be a viable therapeutic strategy. Therefore, we also screened MBB compounds for inhibition of Tm-induced fluorescence in the XBP1s-GFP reporter. Cells were pre-treated with compounds for 2 h before incubating with 0.75 μg/mL of Tm for 12 h. DMSO pretreatment followed by Tm resulted in 2.5-fold GFP induction compared to no Tm addition (Fig. 1f). As a control, pre-treatment with 5 μM of the XBP1 inhibitor mycotrienin II (denoted Myc II) almost completely abolished the Tm-induced GFP fluorescence. Our screen identified three MBB compounds that dampened Tm-induced XBP1s-GFP (Fig. 1d, f). BZI687 (B1) and BZI688 (B2) reduced GFP signal by 80% and 50%, respectively, albeit only when supplied at 50 μM. Soraphen A (B3, denoted SoraA), however, inhibited XBP1s-GFP at 15 μM and 50 μM concentrations (Additional file 2, Fig. S1c). We then tested whether the efficacy of these inhibitors could be enhanced using a low level of Tm. Indeed, with 0.2 μg/mL Tm, these three molecules significantly reduced XBP1s-GFP when supplied at 15 μM and 50 μM (Additional file 2, Fig. S1d).

**Fig. 2:**
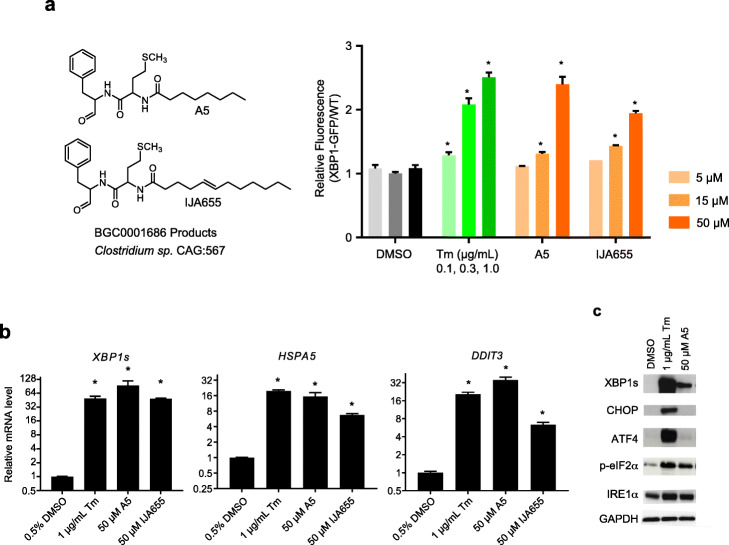
Dipeptide aldehydes induce ER stress and UPR. **a** A5 and its analog IJA655 dose-dependently induce relative XBP1s-GFP. **b** UPR gene mRNA levels were measured in wild-type HT-29 cells treated with Tm, A5, and IJA655, respectively. *ACTB* was used as an internal control, and mRNA levels were normalized to DMSO controls. **c** UPR protein levels were measured in wild-type HT-29 cells treated with DMSO, Tm, and A5 by Western blotting with designated antibodies. In all bar graphs, error bars represent standard errors of the mean from three experimental replicates, and one-way ANOVA was used for statistical analyses (asterisks represent *P* < 0.05)

**Fig. 3:**
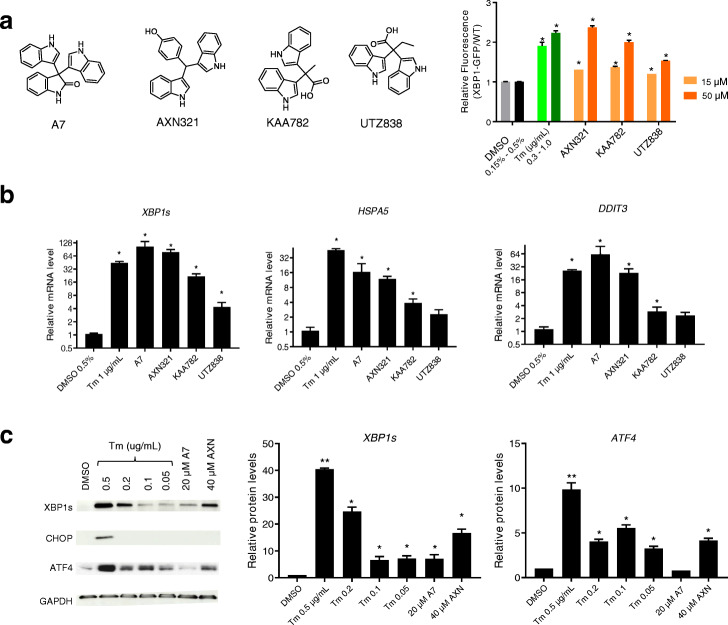
Bisindole-methane derivatives are potent ER stress inducers. **a** Structures of A7 and its analogs were shown and their ability to induce relative XBP1s-GFP were quantified as above. **b** UPR response mRNA levels were measured in wild-type HT-29 cells treated with Tm and bisindole methane derivatives. *ACTB* was used as an internal control, and mRNA levels were normalized to DMSO controls. **c** UPR response protein levels were measured in wild-type HT-29 cells treated with DMSO, Tm, A7, and AXN321 by Western blotting with designated antibodies. The densitometry quantifications were normalized to DMSO treatment, with GAPDH as loading control. The induction of XBP1s by 0.5 μg/mL Tm is estimated by comparing the overexposed western blot of undiluted DMSO control cellular lysate and diluted lysates of Tm treated cellular lysates (see also Additional file 2, Fig. S4b), and inductions by other compound treatments were interpolated by densitometry comparisons with the 0.5 μg/mL treatment. In all bar graphs, error bars represent standard errors of the mean from three experimental replicates, and one-way ANOVA was used for statistical analyses (asterisks represent *P* < 0.05).

To validate the activity of these compounds in wild-type HT-29, we measured mRNA levels in these cells. As expected, Tm induced the spliced-form of XBP1 (*XBP1s*) relative to unspliced *XBP1u* by up to 50-fold (EC_50_ 0.3 μg/mL) compared to DMSO (Additional file 2, Fig. S2a). *HSPA5* and *DDIT3*, two UPR target genes, were induced up to ~ 30-fold (Additional file 2, Fig. S2a, b). Addition of A2 and A6 induced these two transcripts by only 2–3-fold (Additional file 2, Fig. S2b). By contrast, 50 μM A5 or A7 increased both transcript levels by ~ 15 to 30-fold. A5 (*N*-octanoyl-Met-Phe-H, structure shown in Fig. 2a) is an acylated Phe-Met dipeptide aldehyde, whereas A7 is trisindoline (structure shown in Fig. 3a).

Consistently, the inhibitor molecules B1 and B3 decreased Tm-induced expression of *HSPA5* and *DDIT3* (Additional file 2, Fig. S2c). 4μ8C, an IRE1 RNase-selective inhibitor, on the other hand, only reduced *XBP1s* but not *HSPA5* or *DDIT3* (Additional file 2, Fig. S2d). Taken together, our screen identified several MBB compounds with ER stress modulation activities; among these, we chose to focus on the most active molecules in this screen, the activators A5 and A7 and the inhibitor SoraA, for subsequent studies.

### Dipeptide aldehydes induce UPR pathways

A5 is produced by expression of a *Clostridium* non-ribosomal peptide synthase gene cluster BGC0001686 in *E. coli* [34]. Dipeptide aldehydes similar to A5 are commonly made by gut bacteria containing such biosynthetic gene clusters [35]. For instance, IJA655 is co-produced with A5 by BGC0001686 over-expression (personal communication with Voigt lab) [34]. Its acyl chain is 4 carbons longer than that of A5 and harbors an internal double bond (Fig. 2a). Similar to A5, IJA655 induced relative fluorescence in the XBP1s-GFP cell line at 15 μM and 50 μM (Fig. 2a). Both A5 and IJA655 increased the amount of *XBP1s* mRNA (Fig. 2b). However, A5 reduced unspliced *XBP1u* levels by 50% whereas Tm and IJA655 slightly increased *Xbp1u* by about two-fold (Additional file 2, Fig. S3). This result indicates that A5 and IJA655 might have a different effect on transcriptional regulators of XBP. In addition, A5 maximally upregulated *HSPA5* and *DDIT3*, similar to the magnitude of induction by Tm (Fig. 2b) while IJA655 induced these two genes to a lesser extent. Expression of *IRE1α* and *PERK*, encoding two upstream regulators of UPR, are also activated to a lesser extent by IJA655 compared to A5 (Additional file 2, Fig. S3).

To test if dipeptide aldehyde upregulates UPR protein expression levels, we analyzed HT-29 cell extracts by Western blotting (Fig. 2c). 1 μg/mL Tm strongly induced XBP1s, CHOP and ATF4 compared to the DMSO control. We also measured these protein levels in HT-29 cells when exposed to different lower concentrations of Tm (Additional file 2, Fig. S4a). XBP1s and ATF4 induction showed a gradual response to Tm with an EC_50_ of 0.2 μg/mL. The CHOP protein, however, was only detectable when cells were treated with more than 0.5 μg/mL Tm. The low sensitivity of CHOP induction to Tm was not due to the limit of detection of Western blotting, because CHOP in the lysates prepared from 0.5 μg/mL Tm treated HT-29 cells remained detectable when diluted 1:8 (Additional file 2, Fig. S4b). The induction of XBP1s by 0.5 μg/mL Tm is estimated to be 40-fold by comparing the overexposed western blot of undiluted DMSO control cellular lysate and diluted lysates of Tm treated cellular lysates (Additional file 2, Fig. S4b). A5 induced XBP1 to a lesser extent (~ 4-fold less in comparison to Tm treatment), with no CHOP and ATF4 induction (Fig. 2c). However, p-eIF2α and IRE1α were induced by A5 at similar levels to Tm treatment. We hypothesize that mild ER stress caused by A5 could complicate the dynamics and steady-state concentrations of UPR proteins. Taken together, A5 induces ER stress in intestinal epithelial cells.

Dipeptide aldehydes are potent protease inhibitors [34, 35]. We rationalize that A5 induces ER stress by causing misfolded protein accumulation. Therefore, we asked whether inhibiting the activity of a particular class of protease inhibitors leads to UPR activation. To this end, we assayed a panel of 24 protease inhibitors (Additional file 3, Table S2) from three different families (metalloprotease inhibitors, serine/cysteine protease inhibitors, and proteasome inhibitors) for their ability to induce fluorescence in the XBP1s-GFP reporter system (Additional file 2, Fig. S5). Most of the metalloprotease inhibitors we tested did not induce GFP when compared to DMSO, with the exception of TNP-470 and Aladotril. Most serine/cysteine protease inhibitors induced XBP1s-GFP between 1.5- to 3-fold, except for TPCK (Tos-Phe-CH2Cl) and Butabindide (TPPII inhibitor, [36]). Notably, APC-2848 (aka. Mu-Phe-HPh-VSPh), an irreversible cysteine protease inhibitor with a dipeptide vinylsulfone motif, induced fluorescence to a level similar to IJA655. Finally, 3 out of 4 proteasome inhibitors tested induced GFP. These results suggest dipeptide aldehydes potentially activate UPR by inhibiting these proteases.

### Indole-derivatives are potent ER stress inducers

In addition to dipeptide aldehydes, we also identified bisindole derivatives as a different class of activators of ER stress (Fig. 1d, e). A7 is a trisindoline isolated from organic extraction of an *Escherichia fergusonii* (DSM13698) culture grown in MP6 medium (see the “Methods” section)*.* Trisindoline has also been isolated from several other bacterial phyla, including Vibrios, *Rubrivivax*, and Rhodococcus [37–40]. Trisindoline and other bisindole derivatives are presumably formed by condensation of indole-derivatives (indoxyl or isatin) during the bacterial life cycle, or non-enzymatically. Intriguingly, we have detected several other indole derivatives from our collection of commensal bacterial cultures. Specifically, three such molecules extracted from *Porphyromonas uenonis* also induced fluorescence in the XBP1s-GFP reporter (Fig. 3a). Similarly, all molecules triggered the ER-stress response at the transcript level, with AXN321 inducing the highest level of mRNA among the four indole-derivatives (Fig. 3b, 50-fold and 10-fold respectively for *XBP1s* and *HSPA5*). Consistently, AXN321 induced XBP1s and ATF4 moderately, similar to the induction by 0.2 μg/mL. No induction of CHOP was detected at the given concentrations (Fig. 3c). Our findings identified bisindole derivatives as a new class of small molecules with ER stress induction activity.

### Soraphen A prevents tunicamycin induced UPR response

**Fig. 4:**
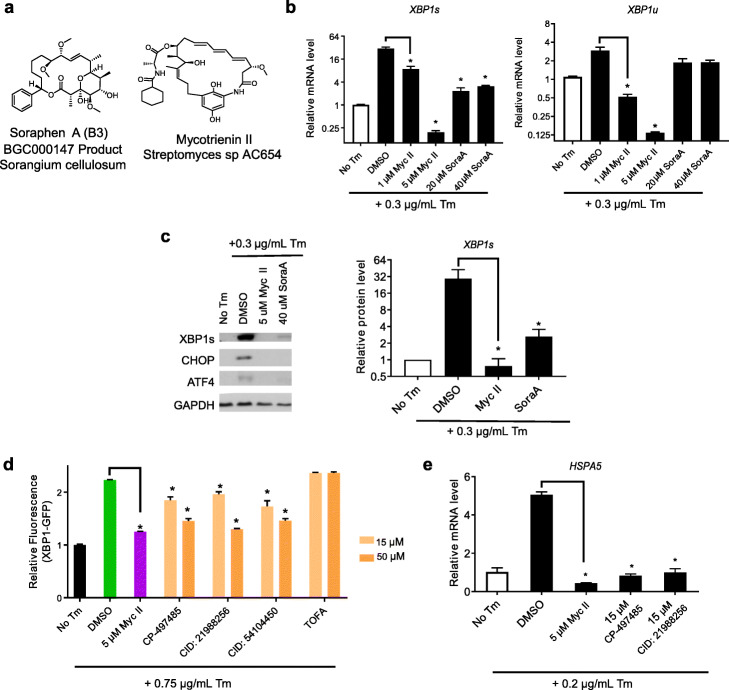
Soraphen A prevents tunicamycin-mediated induction of UPR response. **a** Structures of soraphen A (B3) and Myc II. **b** XBP1s and XBP1u mRNA levels were measured in wild-type HT-29 cells in response to 0.5% v/v DMSO, Myc II, or SoraA pretreatment for 2 h followed by 0.3 μg/mL Tm for 12 h. *ACTB* was used as an internal control, and mRNA levels were normalized to no Tm treatment controls. **c** UPR response protein levels in response to Tm were measured in wild-type HT-29 cells treated with DMSO, Myc II, and SoraA followed by Tm using Western blotting with designated antibodies. Bar graph shows relative protein levels quantified by densitometry as in Fig. 3c. **d** Relative fluorescence of XBP1s-GFP cells in response to 0.75 μg/mL Tm was measured when pre-treated with DMSO, Myc II, or selected ACC inhibitors. **e** *HSPA5* mRNA levels in HT-29 cells in response to 0.2 μg/mL Tm when pretreated with DMSO, Myc II, and ACC inhibitors. In all bar graphs, error bars represent standard error of the mean from three experimental replicates, and one-way ANOVA was used for statistical analyses (asterisks represent *P* < 0.05)

One potent inhibitor of Tm-induced UPR identified in our screen was SoraA, a cyclic polyketide produced by *Sorangium cellulosum* containing a biosynthetic gene cluster (MIBiG BGC000147). Notably, the XBP1 inhibitor Myc II is also a macrocyclic polyketide (structure shown in Fig. 4a). We compared how these two polyketides modulate Tm induction of UPR pathways (Fig. 4b). When the cells were pretreated with 5 μM Myc II followed by Tm, both *XBP1u* and *XBP1s* are below basal levels (by 20-fold and 150-fold respectively, compared to the DMSO control, Fig. 4b). Myc II also prevented Tm induction of XBP1s protein (Fig. 4c). SoraA reduced X*BP1s* and XBP1s activation by 10-fold and 5-fold, respectively, relative to DMSO treatment without affecting the transcript level of *XBP1u* (Fig. 4b, c). In addition to reducing the *XBP1s/XBP1u* ratio, SoraA also decreased the Tm-induced activation of *IRE1α* transcript and proteins (Additional file 2, Fig. S6a and Fig. S6b). These data suggest that SoraA functions predominantly by inhibiting IRE1*α* levels, whereas Myc II inhibits IRE1*α* splicing and XBP1 transcription. Despite these differences, both Myc II and SoraA eliminated ATF4 and CHOP protein activation (Fig. 4c), and *PERK*, *HSPA5*, and *DDIT3* transcript activation induced by Tm (Additional file 2, Fig. S6a).

We examined whether these polyketides could reverse the UPR protein induction when triggered by Tm before inhibitor treatment (Additional file 2, Fig. S6b). Myc II eliminated Tm-induced activation of XBP1s, CHOP and ATF4 when Tm was added 1 h before treatment. SoraA decreased, but did not abolish, XBP1s and CHOP activation by Tm treatment. ATF4 induction could not be reversed by SoraA treatment. These results indicate that SoraA is less able to inhibit UPR response when given after Tm-mediated induction of ER stress has already occurred, suggesting a different mode of action from Myc II. SoraA was reported to inhibit acetyl Co-A carboxylase (ACC) and interfere with fatty acid elongation [41]. Therefore, we characterized a set of ACC inhibitors for their activity to antagonize the effect of Tm-induced UPR response using the XBP1s-GFP assay. Indeed, three out of four AAC inhibitors we tested, with the exception of 5-(Tetradecyloxy)-2-furoic acid (TOFA), reduced Tm-mediated XBP1s-GFP activation (Fig. 4d). Consistently, these AAC inhibitor molecules also reduced *HSPA5* by Tm induction when added as pretreatment (Fig. 4e). These results suggest certain ACC inhibitors could antagonize the UPR response, demonstrating crosstalk between lipid metabolism and ER stress.

### ER stress modulating metabolites induce apoptosis and compromise barrier function

**Fig. 5:**
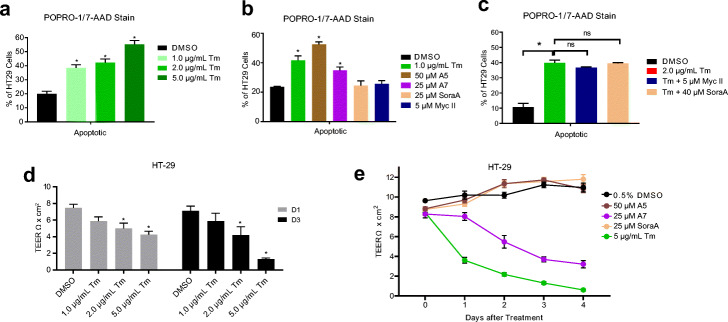
ER stress modulating metabolites induce epithelial cell apoptosis and disrupt barrier function in vitro. **a** Tm induced apoptosis of HT-29 cells after 48 hrs. **b** ER stress activators A5 and A7 induced apoptosis, whereas the ER stress inhibitors Myc II or SoraA had no effect. **c** Pretreatment with Myc II or SoraA did not reduce Tm induced apoptosis. **d** High concentration of Tm decreases the TEER formed by HT-29 cells. **e** A7 decreased TEER after two days of incubation, while neither A5 nor SoraA significantly changed TEER

As mentioned above, prolonged ER stress activation and UPR response can result in apoptosis. We tested these selected metabolites for their effects on HT-29 apoptosis after 2-day compound treatment using the PO-PRO-1/ 7AAD staining kit and FACS analysis. As controls, addition of 1–5 μg/mL Tm induced apoptosis by up to 3-fold (Fig. 5a). Treatment with the ER stress activators A5 and A7 induced apoptosis by 2.5-fold and 1.5-fold, respectively, whereas the ER stress inhibitors Myc II or SoraA had no effect (Fig. 5b). Pretreatment with Myc II or SoraA did not reduce Tm induced apoptosis (Fig. 5c). We hypothesized that induction of cellular apoptosis will disrupt epithelial barrier function, and tested transepithelial electrical resistance (TEER) in HT-29 cells formed in Transwell inserts. Indeed, addition of 2–5 μg/mL Tm decreases the TEER by 30–40% after day 1 and by 40–80% after day 3, respectively (Fig. 5d and Additional file 2, Fig. S7a). Addition of 1 μg/mL is insufficient to reduce TEER significantly, presumably due to the replenishing cellular growth. A7 decreased TEER after two days of incubation, while neither A5 nor SoraA significantly changed TEER (Fig. 5e). Similar results were recapitulated in Caco-2 cells as shown in Additional file 2, Figs. S7b and S7c.

## Discussion

A few soil microbial metabolites and pathogens (e.g., tunicamycin and Shiga toxins) have previously been identified as modulators of the ER stress pathway [28, 32]. We hypothesized that gut microbial metabolites have the capacity to influence inflammatory diseases through ER stress modulation. Here, we have identified gut microbial metabolites that modulate the ER stress pathway. In a screen of 399 MBB compounds, we identified several metabolites that elicit XBP1 activation, and three compounds that inhibit XBP1 activation by Tm. Specifically, we found that acylated dipeptide aldehydes induce that pathway and that Cys/Ser protease inhibition might be a general mechanism for their ER stress activation. Similar to dipeptide aldehydes, bisindole methane derivatives elevated XBP1s-GFP expression and UPR pathway transcripts and protein levels. However, neither dipeptide aldehydes nor bisindole derivatives induced detectable CHOP, one of the apoptosis-related responses observed with Tm activation. On the other hand, the acetyl Co-A carboxylase inhibitor SoraA attenuated the Tm-induced ER stress response when provided prior to Tm treatment. ACC inhibition could prevent Tm from triggering the ER stress response, providing a potential mechanism of action for SoraA. These findings demonstrate that gut microbial products or metabolites have the potential to modulate the ER stress response, and below we describe how these activities could provide mechanistic insights and novel research directions to elucidate the relation of the microbiome to inflammatory diseases.

First, *N*-acylated dipeptide aldehydes moderately induced ER stress. These potent protease inhibitors have in vitro activities against cathepsins B, L, C, and S, and proteasomes [34]. The dipeptide aldehydes are less potent in our endogenous cell-reporter assay. It is possible that when supplied outside of cells, higher concentrations of these molecules would be required to reach inhibitory intracellular concentrations in our GFP assay. Alternatively, a higher concentration may be required to inhibit proteases at levels found in vivo. Dipeptide aldehyde biosynthetic gene clusters were widely found in publicly available human metagenomics data from studies such as the Human Microbiome Project. Gut bacteria produce many varieties of peptide aldehydes that function as protease and proteasome inhibitors [34, 35, 42, 43]. In particular, *Ruminococcus* encoded ruminopeptin is an *N*-acylated Leu-Glu dipeptide aldehyde similar to A5. Proteasome and protease inhibitors impede epithelial restitution in mice or cause gastrointestinal pathology in humans. It is possible that multiple microbially derived molecules synergize with dipeptide aldehydes to function collectively to influence ER stress activation in the gut lumen.

We have also identified trisindoline and several other bisindole methane derivatives that activate ER stress. A variety of similar molecules have been isolated from bacterial sources, including streptindole (from *Streptococcus faecium* IB 37 and *Bacillus subtilis*), vibrindole A (from *Vibrio parahaemolyticus*), arundine and tris(1H-indol-3-yl)methane (from *Vibrio parahaemolyticus* Bio249), as well as arsindoline A and B (from *Aeromonas sp.* CB101) [37, 38, 44–46]. Indole oxidation enzymes such as naphthalene dioxygenase (NDO), multicomponent phenol hydroxylase (mPH), cytochrome P450 monooxygenase, and flavin monooxygenase (FMO) can generate bisindole methanes [47]. Notable activities of bisindole methanes include DNA-damaging properties (streptindole), antibiotic activities (trisindoline), and cytotoxicity against A-549 cell lines (arsindoline B, IC_50_ 22.6 μM). Bisindole methanes also activate peroxisome proliferator-activated receptor γ (PPARγ), retinoic acid receptor, retinoic X receptor, estrogen receptor α, or the aryl hydrocarbon receptor [48–51]. Our finding that bisindole methanes are involved in ER stress activation uncovers a new biological activity for this class of molecules and could contribute to their anti-cancer activities. Particularly, AXN321 (aka DIM-C-pPhOH) acts as an NR4A1 antagonist that inhibits pro-oncogenic pathways in cancer cells [52]. We envision that ER stress inducing activities of these molecules against healthy epithelial cells, as well as interactions with other receptors, could synergistically lead to gut barrier function disruption and tissue inflammation. Identifying the different modes of action between bisindoles and Tm might uncover new targets for IBD therapeutics.

We found that SoraA dampens the UPR response in mammalian cells. We have yet to isolate a gut microbe strain that contains a complete soraphen biosynthetic gene cluster, but we found homologs of the SorA protein in several *Actinoalloteichus* and *Mycobacteria*. We speculate that gut microbes might modulate ER-stress using molecules analogous to SoraA. The ability of this ACC inhibitor to antagonize Tm-induced XBP1s expression adds to the accumulating evidence that the UPR intimately intersects with lipid homeostasis. Membrane stress activates UPR, as was initially observed by induction of BiP/KAR2 in yeast cells grown in inositol depleted medium [53]. In small rodents, obesity stimulates ER stress in liver and adipose cells [2], and exposure to saturated fatty acids leads to phosphorylation of PERK and causes cell death in INS-1 pancreatic β cells [54]. While saturated lipid toxicity can activate ER stress, studies also show that lipid synthesis is required for ER stress resolution [55]. Tm-induced ER stress enhances lipid production by IRE1/XBP1 mediated upregulation of *Dgat2*, *Scd1*, and *Acc2* [56], which is an adaptive response to restore lipid membrane homeostasis. It is reasonable that gut microbes could affect lipid metabolism of the gut epithelium and contribute to the disease progression of IBD patients via UPR dysregulation.

## Conclusions

Our study offers several links between specific classes of gut microbe-derived metabolites and the ER stress response. The diversity of the gut microbiome and the metabolites produced by these bacteria suggests an even greater potential for the contribution of microbial UPR regulation to intestinal epithelial cell ER homeostasis. Leveraging these molecules as potential therapeutics will require optimization of pharmacokinetic/pharmacodynamic and further studies to increase potency [57]. Our findings provide a greater understanding of how microbial metabolism may contribute to the adaptive vs. pathological ER stress response observed in IBD patients.

## Methods

### Cell culture, DNA transfections, and construction of reporter cell lines

HT-29 cells were derived from the ATCC stock/core facility at Broad Institute. Cells were grown in DMEM with high glucose and GlutaMAX™ (Thermo Fisher, 10569044), supplemented with 10% FBS, 100 units/mL penicillin, and 100 μg/mL streptomycin. Cells were treated with tunicamycin (Sigma, T7765) or other compounds solubilized in DMSO. (Tm is composed of a mixture of 4 similar homologs, and a 1 μg/mL mixture solution is approximately equivalent to 1 μM). The XBP1s-GFP reporter cell line was generated from our previous work [58]. HT-29 cells were transfected with the mixture of Cas9-guide RNA complex with repair template dsDNA using Lonza 4D nucleofector with 4D-nucleofector kit (V4XC-2032) according to the manufacturer’s protocol. We used the guide RNA sequences targeting the C-terminal region of XBP1s and the repair template dsDNA containing the C-terminal region of XBP1s and P2A-GFP sequence. We selected the single clones that expressed GFP protein upon Tm treatment using a FACS sorter (Sony SH800) and confirmed the knock-In construct by sequencing. MP6 Media: wheat starch (Fluka 85649, 50 g/L), yeast extract (Difco 0127-08, 15 g/L), KH_2_PO_4_ (0.5 g/L), MgSO_4_ (0.5 g/L), CaCO_3_ (5 g/L).

The human intestinal Caco-2 cell line was obtained from ATCC (HTB-37). Cells were maintained at 37 °C in 95% air-5% CO_2_ atmosphere in Dulbecco’s Modified Eagle’s Medium (DMEM, Thermo Fisher 11885) containing 5.5 mM glucose, 1 mM pyruvate, 100 U/mL penicillin, and 100 μg/mL streptomycin, supplemented with 10% heat-inactivated FBS (FBS-Hyclone Laboratories, Logan, UT, USA).

### XBP1s-GFP assay

XBP1s-GFP cells (7.5 × 10^3^) were seeded in triplicate in 384-well plates and cultured in 25 μL DMEM for 24 h at 37 °C with 5% CO_2_. Stock solutions of Tm and MBB compounds were dissolved in DMSO and added to cells to reach 0.5% DMSO v/v. After 12 h incubation with the compounds, cells were stained with 5 μg/mL Hoechst 33342 (Thermo Fisher, H3570) and imaged with IN Cell Analyzer 6500 HS high content imaging system for DAPI and GFP fluorescence. Fluorescence images were analyzed by CellProfiler to identify cell nuclei and cytoplasm and mean cellular GFP fluorescence was calculated. For XBP1s-GFP inhibition, Tm was added 2 h following compound treatment (unless otherwise noted) and fluorescence was measured after 12 h.

### mRNA real-time quantitative PCR

Total mRNA from HT-29 cells was extracted and reverse transcribed using TaqMan Fast Advanced Cells-to-Ct Kit (Thermo Fisher, A35374) according to the manufacturer’s instructions. Briefly, cells were washed in PBS and lysed for five minutes at room temperature with DNase treatment. Cell lysates (45% v/v for RT reaction) were used for reverse transcription to generate cDNA. 1-2 μL of cDNA was used for Taqman Gene Expression Assays. Expression of mRNA was calculated after normalization to ACTB mRNA. Probes against *XBP1s* (Hs03929085_g1), *XBP1u* (Hs02856596_m1), *IRE1α*(Hs00176385_m1), *PERK* (Hs00984005_m1), *HSPA5* (Hs00607129_gH), *DDIT3* (Hs00358796_g1), and *ACTB* (Hs99999903_m1) were purchased from Thermo Fisher Scientific.

### Western blotting analysis

Cells were harvested in RIPA cell lysis buffer (Thermo Fisher, 89900) and heated for 5 min at 100 °C. Equal quantities of denatured protein samples were resolved on 10% SDS-polyacrylamide gels and were then transferred onto nitrocellulose membranes (Roche, Basel, Switzerland). After blocking with 5% non-fat dry milk in TBS/0.05% Tween-20, the membranes were incubated with a specific primary antibody (1:1000) followed by a horseradish peroxidase-conjugated secondary antibody. Rabbit antibodies against XBP1s (clone D2C1F, #12782), CHOP (D46F1, #5554), ATF4 (D4B8, #11815), IRE1α (14C10, #3294), p-eIF2 (D9G8, #3398), and GAPDH (14C10, #2118) were purchased from Cell Signaling Technologies. Proteins were visualized using ECL reagents (Pierce, Rockford, IL, USA) and analyzed with Fiji software to quantify intensity of the bands. Protein levels were normalized to GAPDH. Expression of XBP1s in the untreated samples is below the limit of detection. For XBP1s quantification, we used overexposed film to quantify the fold difference between untreated samples and the 0.5 μg/mL Tm control. The XBP1s levels from other treatments are then interpolated based on their intensities in comparison with the 0.5 μg/mL sample. In addition, we have used serial dilution to obtain the levels of expected band intensity to verify the quantification is in the right range.

### Detection of HT-29 apoptosis by FACS

A cell apoptosis kit (Thermo Fisher V35123) containing PO-PRO-1 and 7-aminoactinomycin D (7-AAD) was used to detect HT-29 apoptosis. HT-29 cells cultured in 384-well were treated with compounds for 2 days and harvested by 0.25% trypsin. Cells were then incubated with PO-PRO-1 and 7-AAD according to the manufacturer’s instructions. Cells that are PO-PRO-1 positive are apoptotic, both PO-PRO-1 and 7-AAD positive, or only 7-AAD positive, are dead, and neither PO-PRO-1 nor 7-AAD positive are alive. FACS results were analyzed using FlowJo v10 software (FlowJo LLC, Ashland, Oregon, USA).

### Permeability experiments

Permeability of the cell monolayer was monitored at 18 days from seeding by measuring trans epithelial electrical resistance (TEER). HT-29 or Caco-2 cells were seeded on polycarbonate membrane Transwell inserts (4.26 mm membrane diameter, 0.143 cm^2^ growth area, 0.4 μ pore size (Corning 3391, Lowell, MA, USA) at a density of 5 × 10^5^ cells/cm^2^ and allowed to differentiate for 18 days. Medium was regularly changed three times a week. Before and after compound treatment, the integrity of the cell monolayers was monitored using a REMS-96 autosampler (World Precision Instruments, Sarasota FL).

### Statistical analysis

All statistical analyses were performed using the Graphpad Prism statistical software package. Data are expressed as the means ± S.E. Comparisons between groups were performed with one-way ANOVA. In all cases, a *P* value < 0.05 was considered statistically significant.

## Supplementary Information

**Additional file 1:** **Table S1.** List of MBB molecules.

**Additional file 2:** **Supplementary Figures S1-S7.**

**Additional file 3:** **Table S2.** List of protease inhibitors.

**Additional file 4.** Review history.

## Data Availability

The datasets supporting the conclusions of this article are included within the article and its additional files.
