# Supplementary material for: Gut bacterial metabolites modulate endoplasmic reticulum stress
Source: Genome Biol. 2021 Oct 15;22:292. doi: 10.1186/s13059-021-02496-8 (PMC8518294; doi:10.1186/s13059-021-02496-8)
Supplement: Supplementary file 2 — Additional file 2: Supplementary Figures S1-S7. [file 13059_2021_2496_MOESM2_ESM.pdf]

Figure S1

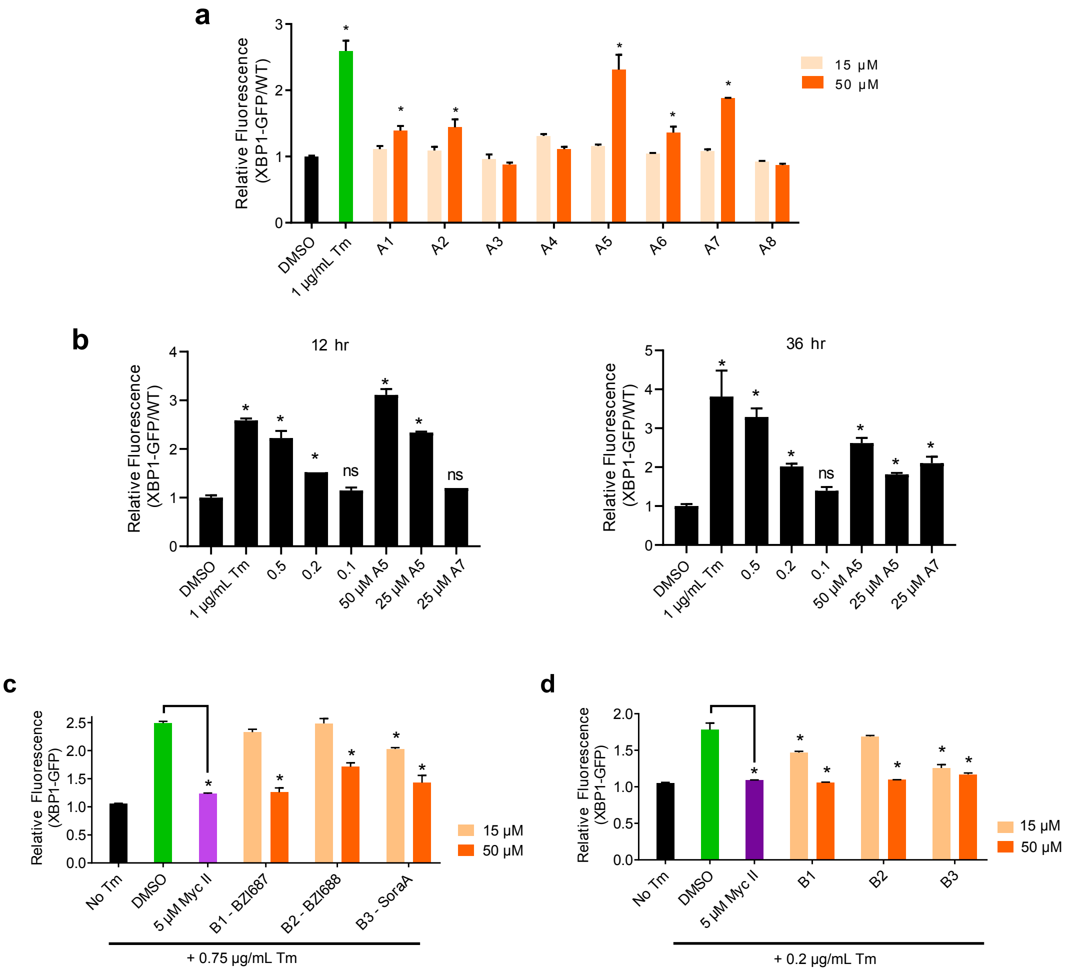
a
Relative Fluorescence (XBP1-GFP/WT)
15 µM
50 µM
DMSO
1 µg/mL Tm
A1
A2
A3
A4
A5
A6
A7
A8
b
12 hr
36 hr
Relative Fluorescence (XBP1-GFP/WT)
ns
DMSO
1 µg/mL Tm
0.5
0.2
0.1
50 µM A5
25 µM A5
25 µM A7
c
Relative Fluorescence (XBP1-GFP)
15 µM
50 µM
No Tm
DMSO
5 µM Myc II
B1 - BZI687
B2 - BZI688
B3 - SoraA
+ 0.75 µg/mL Tm
d
Relative Fluorescence (XBP1-GFP)
15 µM
50 µM
No Tm
DMSO
5 µM Myc II
B1
B2
B3
+ 0.2 µg/mL Tm

Figure S1. Microbiome molecules influence ER stress activity in XBP1s-GFP reporter cells. **a** Eight molecules (A1 - A8) that increased fluorescence in XBP1s-GFP reporter cells from our screen were tested for fluorescence in HT-29 parental cell line (WT) when added. Ratio of the fluorescence in XBP1s-GFP cells to WT when treated with 15 µM and 50 µM molecules were plotted. **b** Various concentrations of Tm and MMB metabolites (A5 and A7) were introduced to XBP1s-GFP for 12 hrs and 36 hrs, and the ratio of the fluorescence relative to the WT cells under same treatments were plotted, respectively. **c** Three MBB molecules (B1 - B3) reduced XBP1s-GFP induction when added in cells 2 hrs before 0.75 µg/mL Tm treatment, indicated by reduced fluorescence compared with DMSO pretreatment. Cell fluorescence was normalized to no Tm treatment. **d** XBP1s-GFP was measured for the indicated molecules which were added to cells 2 hrs before 0.2 µg/mL Tm treatment. In all bar graphs, error bars represent standard error of the mean from three experimental replicates, and one-way ANOVA was used for statistical analyses (asterisks represent $P < 0.05$).

Figure S2

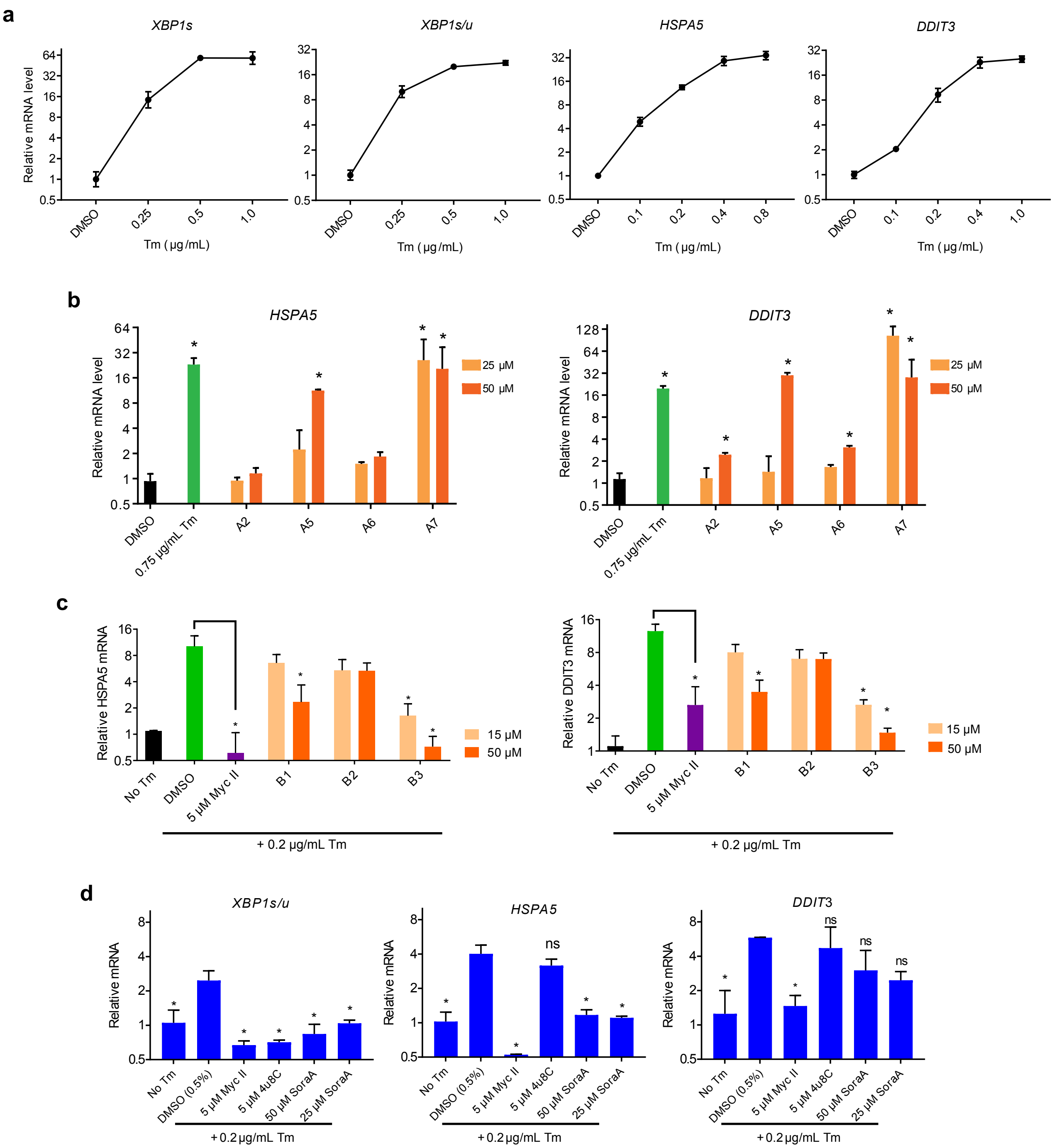
a
XBP1s
XBP1s/u
HSPA5
DDIT3
Relative mRNA level
Tm (μg/mL)
DMSO
b
HSPA5
DDIT3
25 μM
50 μM
0.75 μg/mL Tm
A2
A5
A6
A7
c
Relative HSPA5 mRNA
Relative DDIT3 mRNA
15 μM
50 μM
No Tm
5 μM Myc II
B1
B2
B3
+ 0.2 μg/mL Tm
d
XBP1s/u
HSPA5
DDIT3
Relative mRNA
No Tm
DMSO (0.5%)
5 μM Myc II
5 μM 4u8C
50 μM SoraA
25 μM SoraA
ns
+0.2 μg/mL Tm

Figure S2. Tm and MBB compounds alter expression of UPR genes in HT-29 cells. **a** *XBP1s*, *HSPA5* and *DDIT3* mRNA induction in HT-29 cells were quantified with different Tm concentrations. *ACTB* was used as an internal control, and mRNA levels were normalized to DMSO. **b** XBP1s-GFP inducing molecules were tested for *HSPA5* and *DDIT3* mRNA induction in HT-29 cells. *ACTB* was used as an internal control, and mRNA levels were normalized to DMSO controls. Error bars represent standard error of the mean from three experimental replicates, and one-way ANOVA was used for statistical analyses for comparisons to DMSO controls (asterisks represent $P < 0.05$). **c** *HSPA5* and *DDIT3* mRNA induction by 0.2 µg/mL Tm in HT-29 cells was measured with compound pretreatment, in comparison to 0.5% v/v DMSO pretreatment. *ACTB* was used as an internal control, and mRNA levels were normalized to no Tm treatment controls.Error bars represent standard error of the mean from three experimental replicates, and one-way ANOVA was used for statistical analyses (asterisks represent $P < 0.05$). **d** UPR response gene mRNA levels were measured in wild-type HT-29 cells in response to 0.5% v/v DMSO, MycII, 4u8C, or SoraA pretreatment for 2 hrs followed by 0.2 µg/mL Tm for 12 hrs. *ACTB* was used as an internal control, and mRNA levels were normalized to no Tm treatment controls. One-way ANOVA was used for statistical analyses for multiple comparisons to DMSO control (asterisks represent $P < 0.05$).

Figure S3

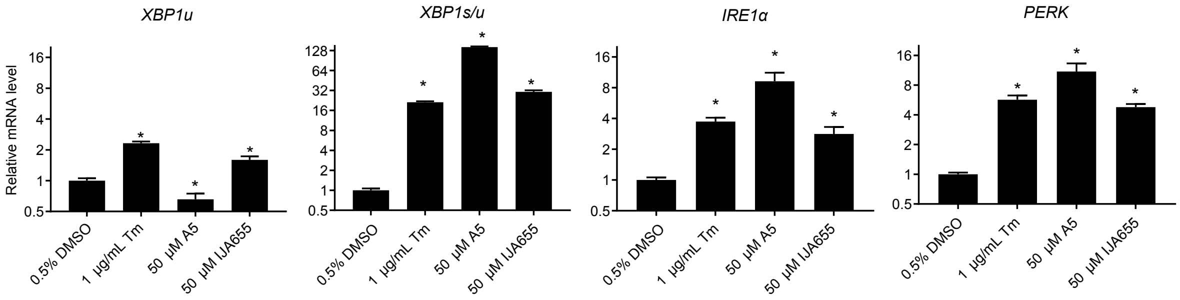

XBP1u
XBP1s/u
IRE1α
PERK
Relative mRNA level
0.5
1
2
4
8
16
32
64
128
*
0.5% DMSO
1 μg/mL Tm
50 μM A5
50 μM IJA655

Figure S3. Dipeptide aldehydes A5 and IJA655 induce UPR response. UPR mRNA levels were measured in wild-type HT-29 cells treated with Tm, A5, and IJA655, respectively. *ACTB* was used as an internal control, and mRNA levels were normalized to DMSO controls. In all bar graphs, error bars represent standard error of the mean from three experimental replicates, and one-way ANOVA was used for statistical analyses (asterisks represent $P < 0.05$).

Figure S4

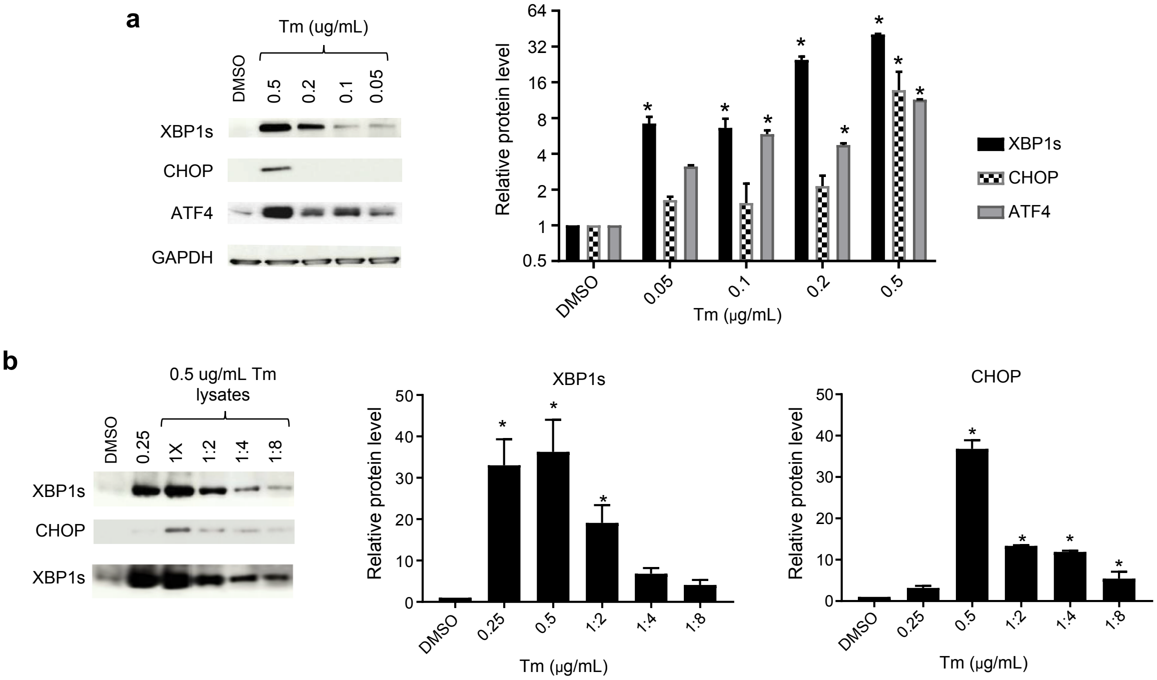

a
Tm (ug/mL)
DMSO
0.5
0.2
0.1
0.05
XBP1s
CHOP
ATF4
GAPDH
Relative protein level
64
32
16
8
4
2
1
0.5
DMSO
0.05
0.1
0.2
0.5
Tm (µg/mL)
XBP1s
CHOP
ATF4
b
0.5 ug/mL Tm
lysates
DMSO
0.25
1X
1:2
1:4
1:8
XBP1s
CHOP
XBP1s
XBP1s
Relative protein level
50
40
30
20
10
0
DMSO
0.25
0.5
1:2
1:4
1:8
Tm (µg/mL)
CHOP
Relative protein level
Tm (µg/mL)

Figure S4. Tm induces expression of UPR protein in HT-29 cells. **a** UPR protein levels were measured in wild-type HT-29 cells treated with different concentrations of Tm. The densitometry quantifications normalized to DMSO treatment are shown in the right panel, with GAPDH as a loading control. Expression of XBP1s and CHOP inthe untreated samples are below the limit of detection. For XBP1s quantification, we used overexposed film to quantify the fold difference between untreated samples and the 0.5 ug/mL Tm control, and interpolated protein expressions for the other treatments by referencing to the 0.5 ug/mL Tm control. **b** Lysates of wild-type HT-29 cells treated with 0.5 µg/mL Tm were serially diluted and probed for XBP1s and CHOP protein levels together with undiluted lysates from DMSO control and 0.25 ug/mL Tm treatment by Western blotting. The overexposed film for XBP1s is shown at the bottom. The densitometry quantifications normalized to DMSO treatment are shown in the right panel, with GAPDH as loading control. Error bars represent standard errors of the mean from three experimental replicates, and one-way ANOVA was used for statistical analyses (asterisks represent $P < 0.05$).

Figure S5

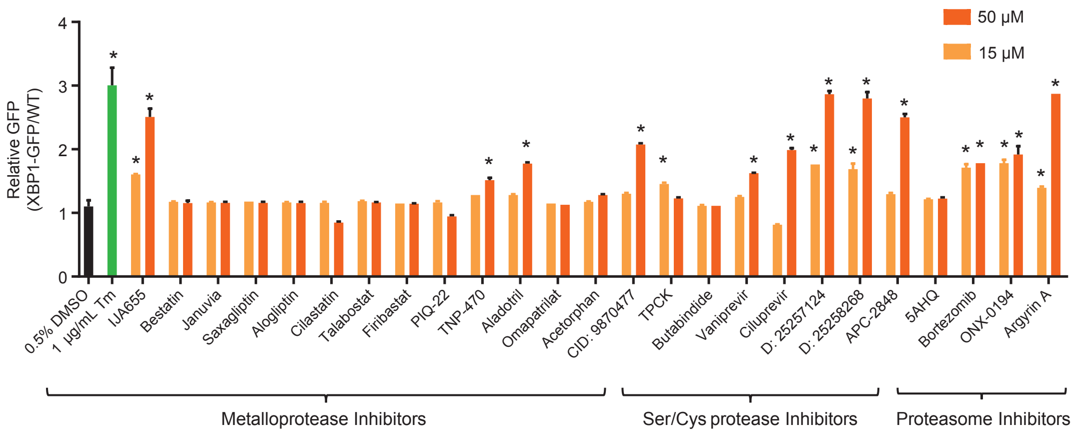
50 µM
15 µM
Relative GFP (XBP1-GFP/WT)
0
1
2
3
4
0.5% DMSO
1 µg/mL Tm
IJA655
Bestatin
Januvia
Saxagliptin
Alogliptin
Cilastatin
Talabostat
Firibastat
PIQ-22
TNP-470
Aladotril
Omapatrilat
Acetorphan
CID: 9870477
TPCK
Butabindide
Vaniprevir
Ciluprevir
D: 25257124
D: 25258268
APC-2848
5AHQ
Bortezomib
ONX-0194
Argyrin A
Metalloprotease Inhibitors
Ser/Cys protease Inhibitors
Proteasome Inhibitors

Figure S5. A panel of protease inhibitors activates XBP1s-GFP induction when supplied at 15 and 50 µM. Ratio of green fluorescence in the reporter lines to parental HT-29 (XBP1s-GFP/WT) were plotted. Tm and IJA655 were positive controls, and the proteases were grouped into three categories (brackets under the bar graph). Error bars represent standard errors of the mean from three experimental replicates, and one-way ANOVA was used for statistical analyses (asterisks represent $P < 0.05$).

Figure S6

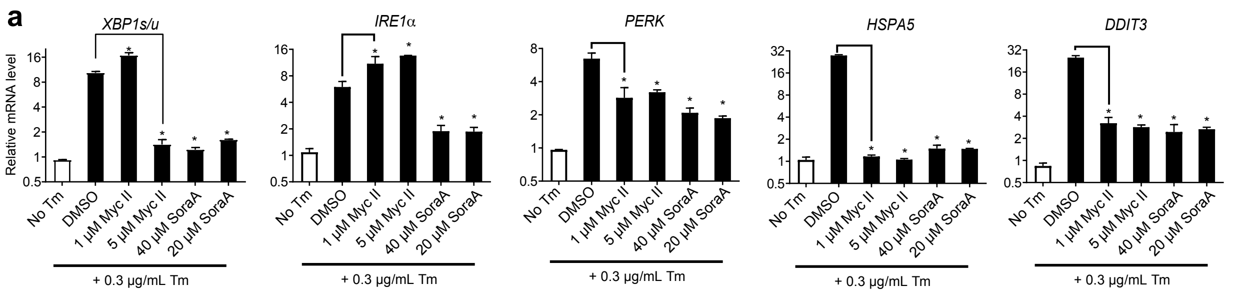

a
XBP1s/u
IRE1α
PERK
HSPA5
DDIT3
Relative mRNA level
No Tm
DMSO
1 µM Myc II
5 µM Myc II
40 µM SoraA
20 µM SoraA
+ 0.3 µg/mL Tm

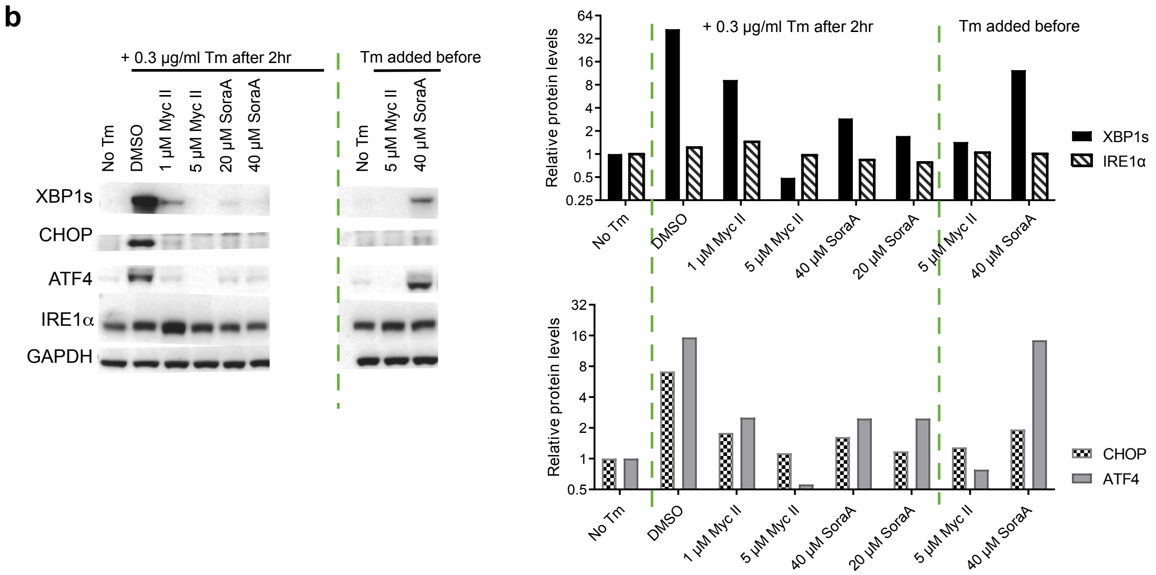

b
+ 0.3 µg/ml Tm after 2hr
Tm added before
No Tm
DMSO
1 µM Myc II
5 µM Myc II
20 µM SoraA
40 µM SoraA
XBP1s
CHOP
ATF4
IRE1α
GAPDH
Relative protein levels
XBP1s
IRE1α
CHOP
ATF4

Figure S6. Soraphen A pretreatment represses UPR induced by Tm. **a** UPR mRNA induction by 0.3 µg/mL Tm in HT-29 cells was measured with Myc II or SoraA pretreatment, in comparison to 0.5% v/v DMSO pretreatment. *ACTB* was used as an internal control, and mRNA levels were normalized to no Tm treatment controls. Error bars represent standard error of the mean from two experimental replicates, and one-way ANOVA was used for statistical analyses for comparisons to the DMSO controls (asterisks represent $P < 0.05$). **b** UPR protein in HT-29 cells was measured with Myc II or SoraA added 2 hrs before 0.3 µg/mL Tm treatment or added 1hr after Tm treatment. 0.5% v/v DMSO treatment was used as solvent controls. GAPDH was used as an internal control, and densitometry quantifications were normalized to no Tm treatment controls and plotted on the right panels.

Figure S7

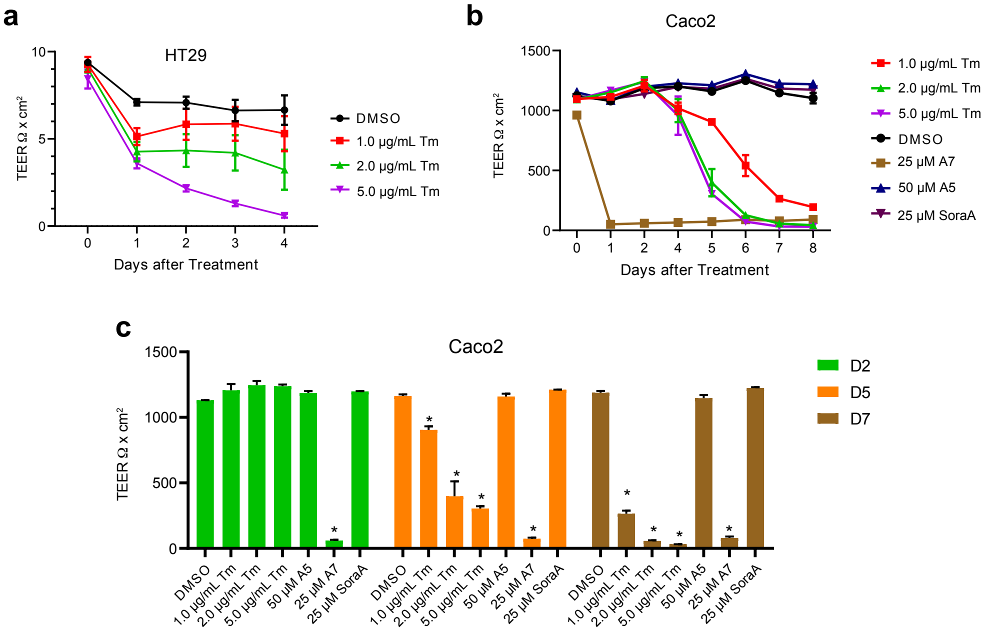

a
HT29
TEER Ω x cm²
Days after Treatment
DMSO
1.0 µg/mL Tm
2.0 µg/mL Tm
5.0 µg/mL Tm
b
Caco2
TEER Ω x cm²
Days after Treatment
1.0 µg/mL Tm
2.0 µg/mL Tm
5.0 µg/mL Tm
DMSO
25 µM A7
50 µM A5
25 µM SoraA
c
Caco2
TEER Ω x cm²
D2
D5
D7
DMSO
1.0 µg/mL Tm
2.0 µg/mL Tm
5.0 µg/mL Tm
50 µM A5
25 µM A7
25 µM SoraA

Figure S7. ER stress modulating metabolites disrupt barrier function in vitro. **a** Tm dose-dependently reduced TEER formed by HT-29 cells in transwells over time. **b** Tm and A7 decreased TEER formed by Caco-2 cells, while neither A5 nor SoraA significantly changed TEER. **c** Comparison of TEER formed by Caco-2 cells after day 2, day 5 and day 7 of compound treatment. Error bars represent standard error of the mean from two experimental replicates, and one-way ANOVA was used for statistical analyses for comparisons to the DMSO controls (asterisks represent $P < 0.05$).
