# Supplementary material for: Gut bacterial metabolites modulate endoplasmic reticulum stress
Source: Genome Biol. 2021 Oct 15;22:292. doi: 10.1186/s13059-021-02496-8 (PMC8518294; doi:10.1186/s13059-021-02496-8)
Supplement: Supplementary file 4 — Additional file 4. Review history. [file 13059_2021_2496_MOESM4_ESM.docx]

Review History

**First round of review**

**Reviewer 1**

**Are you able to assess all statistics in the manuscript, including the appropriateness of statistical tests used?** Yes, and I have assessed the statistics in my report.

**Comments to author:**

In the article "Gut bacterial metabolites modulate endoplasmic reticulum stress", Ke et. al created a library of microbial derived metabolites and screened for their effect on ER stress using a reporter cell line that they engineered by CRISPR. Their output was on the mRNA and protein levels. They linked the metabolites with inflammation and colitis on the molecular level. These authors are clearly experts in this field and present an impressive and valuable study.
The authors studied the microbial metabolites that interact with ER stress signaling, the dysregulation of which has been implicated in intestinal inflammation. The author screened the microbiome box (MBB), a library of 399 chemical compounds relevant to the gut microbiome and IBD. They screened a compound library for modulators of ER stress and identified both inducers and suppressors of this response. They found both ER stress response inducers (acylated dipeptide aldehydes and bisindole methane derivatives) and suppressors (soraphen A), and characterized their activities on ER stress gene transcription and translation. The authors further validated their screening results on the reporter cell line by mRNA measurements. The genes and proteins associated with the ER stress response were studied. They further demonstrated that these molecules modulate the ER stress pathway through protease inhibition or lipid metabolism interference. This study shortlisted the metabolites which could play important roles in gastrointestinal conditions and are linked with the ER stress signaling.
To note, the article includes a comprehensive and very clear introduction to the topic as well as the motivation and the implications of the reported study.
[Minor comment on the introduction- at the end of the introduction - "we aimed" makes more sense to me than "we aim", as this study is already reported. ]
This study, in my opinion, can be accepted as is. It is very comprehensive and adds a lot to our (currently poor) understanding on microbial-derived molecules that affect the host, especially those that are relevant to IBD.
If possible, a few of the following additions could be added and will strengthen this report. All relate to phenotypic validation.
Experiment suggestions:
1.     To study the effect of the molecules A5, A7 and B3 in vivo, especially their effects on mucin and antimicrobial protein production, which are linked to ER proteostasis
2.     Imaging to see the phenotypic effect (eg. Apoptosis) of these molecules, even only in vitro.
3.     To study the inflammatory markers in vitro and in vivo
4.     Since, Xbp1 deletion in mouse intestinal epithelial cells resulted in Paneth and goblet cell apoptosis, spontaneous ileal inflammation and increased sensitivity to dextran sodium sulfate (DSS)-induced colitis can be measured. Such parameters can be studied in vivo with these molecules.
5.     Perhaps a straightforward experiment evaluating the epithelial barrier effects of the relevant microbial produced molecules by measuring transepithelial electrical resistance.

**Reviewer 2**

**Are you able to assess all statistics in the manuscript, including the appropriateness of statistical tests used?** No, I do not feel adequately qualified to assess the statistics.

**Comments to author:**

The authors present an interesting report where they identify metabolites produced by the gut microbiome that can module UPR signaling in mammalian cells. This is a great starting point for a detailed mechanistic study that could potentially be very impactful in the microbiome field. However, the authors provide somewhat limited follow-up on the top candidates, investigating the compounds' effect of some key UPR signaling steps and conducting initial exploration into the compounds' mechanisms of action, but their work in both these areas could be expanded. Further, the authors speculate that these compounds may play a part in microbiome-induced UPR signaling, but provide no evidence of this, and do not even mention how the various concentrations tested here compare to what can be found in vivo. Therefore I think this work is more suited to a more specialized journal.

-S1: 25uM A7 induces significant expression of HSPA5 and DDIT3, but 15uM A7 does not lead to increased fluorescence in cells carrying the Xbp1s-GFP reporter. Does 25uM A7 lead to increased fluorescence in these cells?

-S2b, 2c: How are the band intensities calculated? I cannot see bands from the untreated samples for several genes, so I'm not sure how any other band intensities are normalized to the untreated control.

-mycotrienin II is also a translation inhibitor, which complicates its usage in assays where the readout involves protein synthesis (e.g., the GFP reporter, WBs in Fig. 4c)

-Figure 2c: it is surprising that 50uM A5 induces increased DDIT3 expression and increased p-eIF2a levels, but does not lead to increased ATF4 or CHOP levels, as these transcripts are preferentially translated when there are high levels of p-eIF2a. The authors do not comment on this at all.

-Xbp1s mRNA levels should be normalized to total Xbp1 transcripts to better assess changes in IRE1a RNase activity specifically.

-More details are needed in the methods section to describe the construction of the GFP reporter line (guide RNA sequences, primer sequences, etc.)

Minor notes:
Page 7, line 53; Figure reference error - the structure of CAY146/B3 is not presented in Fig. 1d.
Page 8, lines 18 and 22; BGC0001686 or BGC000186?
Page 8, line 42: no figure referenced for IRE1a and PERK expression.
Page 10, line 58 - there is no figure 5.
-consistency with naming compounds would make it easier for the reader to follow an abbreviation-heavy paper (e.g., referring to Soraphen A as B3 or Sor in different figures)
-page 19, line 44 (methods section) - were 7.5e4 cells seeded in 1 well of a 384-well plate?

**Authors Response**

**Point-by-point responses to the reviewers’ comments:**

Reviewer #1: In the article "Gut bacterial metabolites modulate endoplasmic reticulum stress", Ke et. al created a library of microbial derived metabolites and screened for their effect on ER stress using a reporter cell line that they engineered by CRISPR. Their output was on the mRNA and protein levels. They linked the metabolites with inflammation and colitis on the molecular level. These authors are clearly experts in this field and present an impressive and valuable study.
The authors studied the microbial metabolites that interact with ER stress signaling, the dysregulation of which has been implicated in intestinal inflammation. The author screened the microbiome box (MBB), a library of 399 chemical compounds relevant to the gut microbiome and IBD. They screened a compound library for modulators of ER stress and identified both inducers and suppressors of this response. They found both ER stress response inducers (acylated dipeptide aldehydes and bisindole methane derivatives) and suppressors (soraphen A), and characterized their activities on ER stress gene transcription and translation. The authors further validated their screening results on the reporter cell line by mRNA measurements. The genes and proteins associated with the ER stress response were studied. They further demonstrated that these molecules modulate the ER stress pathway through protease inhibition or lipid metabolism interference. This study shortlisted the metabolites which could play important roles in gastrointestinal conditions and are linked with the ER stress signaling.
To note, the article includes a comprehensive and very clear introduction to the topic as well as the motivation and the implications of the reported study.

Thank you for your enthusiasm for our manuscript.

[Minor comment on the introduction- at the end of the introduction - "we aimed" makes more sense to me than "we aim", as this study is already reported.]

We have made the adjustment in the text accordingly (line 103).

This study, in my opinion, can be accepted as is. It is very comprehensive and adds a lot to our (currently poor) understanding on microbial-derived molecules that affect the host, especially those that are relevant to IBD.
If possible, a few of the following additions could be added and will strengthen this report. All relate to phenotypic validation.

Experiment suggestions:
1. To study the effect of the molecules A5, A7 and B3 in vivo, especially their effects on mucin and antimicrobial protein production, which are linked to ER proteostasis

There is limited current understanding of the pharmacokinetic/pharmacodynamic (PK/PD) activity of these ER stress modulation molecules. We have added the following sentence in the discussion to emphasize this point: “Leveraging these molecules as potential therapeutics will require optimization of pharmacokinetic/pharmacodynamic and further studies to increase potency [(*1*)](https://paperpile.com/c/0cIm9a/qV3gS).”

In lieu of a mouse experiment, we focused our efforts to address the phenotypic effect *in vitro* as described in point 2, 3 and 5 below.

2. Imaging to see the phenotypic effect (eg. Apoptosis) of these molecules, even only in vitro.

We used the PO-PRO-1/7AAD staining kit and FACS analysis to measure apoptosis of HT29 cells after 2-day compound treatment. Addition of 1-5 ug/mL Tm induced apoptosis (see below, Figure 5a). Treatment with the ER stress activators A5 and A7 also induced apoptosis, whereas the ER stress inhibitors Mycotrienine II or SoraA had no effect (Fig. 5b). Pretreatment with Mycotrienine II or SoraA did not reduce Tm induced apoptosis (Fig. 5c). These data have been added to the main manuscript.

3. To study the inflammatory markers in vitro and in vivo

To assess inflammatory markers in vitro, we stimulated HT29 cells with 100 ng/mL TNFa and 20 ng/mL IFN-g and measured CD40 mRNA with or without the ER stress modulators. There was no difference in effect with the addition of these molecules, therefore we have not included these data in the manuscript.

4. Since, Xbp1 deletion in mouse intestinal epithelial cells resulted in Paneth and goblet cell apoptosis, spontaneous ileal inflammation and increased sensitivity to dextran sodium sulfate (DSS)-induced colitis can be measured. Such parameters can be studied in vivo with these molecules.

As described in point 1, given the challenges with PK/PD *in vivo*, we focused on the above *in vitro* experiments.

5. Perhaps a straightforward experiment evaluating the epithelial barrier effects of the relevant microbial produced molecules by measuring transepithelial electrical resistance.

Thanks for the suggestion. We tested transepithelial electrical resistance (TEER) in HT29 cells and found that 2-5 ug/mL Tm was necessary for decreasing the TEER formed by these cells, and that A7 decreased TEER after two days of incubation, while neither A5 nor SoraA significantly changed TEER (new Fig. 5d,e). These results were recapitulated in CaCO2 cells as shown in Fig. S6.

**Fig. 5**

Reviewer #2: The authors present an interesting report where they identify metabolites produced by the gut microbiome that can module UPR signaling in mammalian cells. This is a great starting point for a detailed mechanistic study that could potentially be very impactful in the microbiome field. However, the authors provide somewhat limited follow-up on the top candidates, investigating the compounds' effect of some key UPR signaling steps and conducting initial exploration into the compounds' mechanisms of action, but their work in both these areas could be expanded. Further, the authors speculate that these compounds may play a part in microbiome-induced UPR signaling, but provide no evidence of this, and do not even mention how the various concentrations tested here compare to what can be found in vivo. Therefore I think this work is more suited to a more specialized journal.

Thank you for reviewing our manuscript and providing many suggestions. We have addressed your question in the following section and made modifications accordingly to the manuscript, we hope that you will agree our changes improve the depth of the work.

-S1: 25uM A7 induces significant expression of HSPA5 and DDIT3, but 15uM A7 does not lead to increased fluorescence in cells carrying the Xbp1s-GFP reporter. Does 25uM A7 lead to increased fluorescence in these cells?

We tested 25uM A7 and found minimal increase of XBP1s-GFP in the reporter after 12 hr and 2-fold induction of GFP after 36 hr treatment (Fig. S1c).

The correlation between XBP1s-GFP and mRNA transcripts are not linear. The control, Tm, induced XBP1s splicing up to 50-fold compared to DMSO treated cells, but only induced the fluorescent reporter 2.5-fold. This could be due to lower XBP1s-GFP expression from the HT29 chromosomes potentially masked by background fluorescence. (We added this explanation in the text, line 150). In addition, *HSPA5* and *DDIT3* mRNA levels could have different temporal dynamics compared with GFP protein folding or they could be affected by transcription factors in addition to XBP1s. Using primary intestinal monolayers, our lab has expanded on the complexities of this pathway by performing a genome-wide CRISPR screen paired with single-cell RNA sequencing and identified numerous genes involved in regulating this complex pathway [(*2*)](https://paperpile.com/c/0cIm9a/Ikqm).

-S2b, 2c: How are the band intensities calculated? I cannot see bands from the untreated samples for several genes, so I'm not sure how any other band intensities are normalized to the untreated control.

We apologize for the lack of clarity. We used FIJI software for quantification of the intensity of the bands and normalized protein levels to GAPDH. Expression of these proteins in the untreated samples is difficult to detect, however. For these faint bands, we used overexposed film to quantify the fold difference between untreated samples and the 0.5 ug/mL Tm control. The other bands’ intensities are then estimated based on their comparison with the 0.5 ug/mL sample. In addition, we have used serial dilution to obtain the levels of expected band intensity to verify the quantification is in the right range. We have added these details to the Western blotting analysis methods section and included the overexposed film in Fig. S2c (see below).

-mycotrienin II is also a translation inhibitor, which complicates its usage in assays where the readout involves protein synthesis (e.g., the GFP reporter, WBs in Fig. 4c)

We also considered this possibility and to make sure there was no effect, we tested the expression of several proteins in response to 12 hr treatment of Myc II, and the protein levels of IRE1a and GAPDH were not affected (Fig. S5b). In addition, we have tested the effect of Myc II on a HT29 line constitutively expressing GFP (SL006), we did not observe reduction of GFP fluorescence after 24 hr incubation at the concentration we have used in our assays (see data below from Fig. S4d). In addition, we have tested an IRE1a nuclease inhibitor (4u8C). This molecule should not inhibit protein translation and it inhibited *XBP1s* induced by Tm to a similar extent as MycII, but did not inhibit *HSPA5* or *DDIT3*.

-Figure 2c: it is surprising that 50uM A5 induces increased DDIT3 expression and increased p-eIF2a levels, but does not lead to increased ATF4 or CHOP levels, as these transcripts are preferentially translated when there are high levels of p-eIF2a. The authors do not comment on this at all.

Thank you for this comment. As mentioned above, the correlation between mRNA transcripts and protein levels are not linear. For instance, 0.2 ug/mL Tm induced robust DDIT3 mRNA expression, but this did not translate to detectable CHOP protein levels (Fig. S2b). In addition, 50 µM A5 only induced a weaker UPR response compared to Tm at protein levels (measured by XBP1s), and its potency is equivalent to ~0.1- 0.2 µg/mL Tm. ER stress resolution in response to mild ER stress could further complicate the dynamics and steady-state concentrations of UPR proteins. We clarified this in the text: “We hypothesize that mild ER stress caused by A5 could complicate the dynamics and steady state concentrations of UPR proteins.” (lines 202-203).

-Xbp1s mRNA levels should be normalized to total Xbp1 transcripts to better assess changes in IRE1a RNase activity specifically.
We initially intentionally kept *Xbp1u* and *Xbp1s* transcripts separate to better assess the effect on *Xbp1* transcription regulation. Per your suggestion, we also have included the *Xbp1s/u* ratio in the supplemental figures (Fig S2a, S3a, S4d, S5a).

-More details are needed in the methods section to describe the construction of the GFP reporter line (guide RNA sequences, primer sequences, etc.)

We agree that more information is needed. The method for the GFP reporter construction has recently been published in You et al. 2020. We have added the reference to this in the method session.

Minor notes:
Page 7, line 53; Figure reference error - the structure of CAY146/B3 is not presented in Fig. 1d.

Thank you for catching this! We have corrected the error and referred to its structure in Fig. 4.

Page 8, lines 18 and 22; BGC0001686 or BGC000186?

We have corrected the typo in line 181 to “BGC0001686”.

Page 8, line 42: no figure referenced for IRE1a and PERK expression.

We have added the reference in line 190 to Fig. S3a.

Page 10, line 58 - there is no figure 5.
-consistency with naming compounds would make it easier for the reader to follow an abbreviation-heavy paper (e.g., referring to Soraphen A as B3 or Sor in different figures)

We referred to Soraphen A as “B3” in the initial screen (lines 166-167) to keep consistent with other compound hits in Fig.1. As you recommended, we have abbreviated that to “SoraA” in all the other figures throughout the manuscript after its introduction from the screen.

-page 19, line 44 (methods section) - were 7.5e4 cells seeded in 1 well of a 384-well plate?

Thank you! We have corrected the typo in line 500 to “7.5 x 10^3^”.

References

1.	[D. R. Plichta, D. B. Graham, S. Subramanian, R. J. Xavier, Therapeutic Opportunities in Inflammatory Bowel Disease: Mechanistic Dissection of Host-Microbiome Relationships. *Cell*. **178**, 1041–1056 (2019).](http://paperpile.com/b/0cIm9a/qV3gS)

2.	You K, Wang L, Chou CH, Liu K, Nakata T, Jaiswal A, Yao J, Lefkovith A, Omar A, Perrigoue JG, Towne JE, Regev A, Graham DB, Xavier RJ. QRICH1 dictates the outcome of ER stress through transcriptional control of proteostasis. Science. 2021;371, eabb6896.

**Second round of review**

**Reviewer 2**

The authors addressed all my specific concerns, though I still have a major problem with Fig 2c. I just don't think it's appropriate to show a blank western blot in which you can't see any bands in any lanes and then have a table with band intensity quantification next to it. The figure needs to be reworked.

**Authors Response**

• We have now clearly stated in the figure legend and the main results the details about the Western blot quantification
